# A 3D-polyphenylalanine network inside porous alumina: Synthesis and characterization of an inorganic–organic composite membrane

**DOI:** 10.3762/bjnano.11.78

**Published:** 2020-06-17

**Authors:** Jonathan Stott, Jörg J Schneider

**Affiliations:** 1 Technische Universität Darmstadt, Fachbereich Chemie, Eduard-Zintl-Institut für Anorganische und Physikalische Chemie, Alarich-Weiss Str. 12, 64287 Darmstadt, Germany; 2 Nanoscience for life GmbH & Co. KG, Regerstr. 1, 65193 Wiesbaden, Germany

**Keywords:** alumina, composite membrane, polymer-based sorbent, polyphenylalanine, porous alumina membranes, surface-initiated ring-opening polymerization (SI-ROP)

## Abstract

Surface functionalization of porous materials allows for the introduction of additional functionality coupled with high mechanical stability of functionalized inner pores. Herein, we investigate the surface-initiated ring-opening polymerization (SI-ROP) of phenylalanine-*N*-carboxyanhydride (PA-NCA) in porous alumina membranes (ALOX-membranes) with respect to different solvent mixtures (tetrahydrofuran (THF) and dichloromethane (DCM)). It was found that increasing the volume fraction of DCM leads to an increasing amount of fibrillar polymer structures within the porous ALOX-membrane. A three-dimensional fibrillar network with intrinsic porosity was formed in DCM, whereas in THF, a dense and smooth polypeptide film was observed. A post-treatment with a mixture of chloroform and dichloroacetic acid leads to rearrangement of the morphology of the grafted polymer films. The analysis by scanning electron microscopy (SEM), near-infrared spectroscopy (NIR) and contact angle measurements (CA) reveals a change in morphology of the grafted polymer films, which is due to the rearrangement of the secondary structure of the polypeptides. No significant loss of the surface-grafted polypeptides was determined by thermogravimetric (TG) measurements, which indicates that the change in morphology of the polymer films is solely a result of a conformational change of the surface-grafted polypeptides. Furthermore, adsorption of a test analyte (chloroanilic acid) was investigated with respect to different polymer functionalization schemes for reversed-phase solid phase extraction applications. The adsorption capability of the functionalized composite membrane was increased from 16.7% to 38.1% compared to the native ALOX-membrane.

## Introduction

Porous materials with functional surfaces are a topic of immense interest in science and technology. The high surface area and high mechanical stability of such materials allows for applications in many fields such as membrane separation [1–5] or adsorbent materials [1,6–8]. The combination of inorganic porous supports (such as alumina and a variety of synthetic polypeptides) that can be grafted onto such surfaces is also promising, especially with respect to medical and analytical applications. The most common route to graft polypeptides on surfaces is the surface-initiated ring-opening polymerization (SI-ROP) of α-aminoacid-*N*-carboxyanhydrides (NCAs). Dense polypeptide films consisting of poly(γ-benzyl-ʟ-glutamate), polylysine or polyphenylalanine with thickness in the range between several nanometers to over 100 nanometers were realized on flat surfaces [9–14]. Whitesell et al. first described a SI-ROP of alanine-NCA and phenylalanine-NCA on flat surfaces [15]. In the last decades poly(γ-benzyl-ʟ-glutamate) has become the most prominent polypeptide with respect to SI-NCA-polymerization [10,12–13,16]. Curved surfaces of porous materials were also functionalized by SI-ROP of NCAs [17–18]. These functionalization schemes of porous support materials improve the separation of ions or chiral molecules and can act as supported catalytic materials for oligomerization [19–21]. Films of poly(γ-benzyl-ʟ-glutamate) were surface-grafted within nanoporous anodic alumina (AAO) by surface-initiated polymerization of poly(γ-benzyl-ʟ-glutamate)-NCA [22–23].

Three-dimensional fibrillar networks consisting of supramolecular assemblies of oligomers or polypeptides have attracted significant attention in the last decades. Due their secondary structures (α-helix or β-sheet), polypeptides form macromolecular block segments, which can undergo a self-assembly process under certain circumstances [24–25]. The intramolecular interactions between these macromolecules as well as the mechanisms of gel formation have been studied in detail. Hydrophilic gels with functional side groups enable stimuli-responsive hydrogels with interesting properties with regards to drug release systems. A drawback of these gels is their low mechanical stability, which can however be improved by chemical crosslinking of the gel structures [26–27]. A three-dimensional fibrillar network due photo-crosslinking gelation of poly(γ-benzyl-ʟ-glutamate-co-allylglycine) was achieved by Vacogne et al. These authors have demonstrated that the network-like structure remains intact after deprotection of the side chain. This enables formation of pH-responsive hydrogels [28]. Stable, thermoresponsive organo-gels consisting of polybenzylglutamate-α-helices were studied by Niehoff et al. [29]. Gelated polybenzylglutamate molecules with molecular weight from approximately 7000 to 100 000 g/mol were obtained from hot toluene consisting of a stable fibrillar network. The aggregation of bundles of pBzG_338_-α-helices with a thickness of 1.5 nm and a length of 50.7 nm results in a three-dimensional network with much higher dimensions in the range of micrometers. It was proposed that additional "crosslinking" due to branching and rejoining of fibrillar supramolecular aggregates occurs. Nevertheless "end-to-end" and "side-to-side" interactions of the pBzg-α-helices are essential for the formation of fibrillar networks. Supported hydrogels that were attached to a porous substrate have been reported by Fores et al. The authors realized peptide hydrogels in a porous melamine foam for use in continuous flow chemistry [30]. Other polyelectrolytes such as poly(acrylic acid) were prepared using an ozone-induced grafting process for cellulose fibers [31]. Hydrophobic foams of poly(γ-benzyl-ʟ-glutamate-co-ʟ-phenylalanine) were fabricated by a template method reported by Cui et al. [32].

In this study, we functionalize porous alumina substrates to induce the formation of three-dimensional supported organo-gels within their porous architecture to study a new inorganic–organic composite material. Such a surface-grafted gel might be interesting in membrane separation technology or supported drug release/adsorption systems. We investigate the ability of polyphenylalanine to form organo-gels in situ within a porous inorganic environment with respect to different volume fractions of DCM in the solvent mixture. We chose phenylalanine as an aromatic and hydrophobic amino acid to achieve a good interaction with slightly water-soluble compounds (e.g. chloroanilic acid) in aqueous solutions. Due the lack of amino groups in the side chain of phenylalanine, no further protection groups are needed for NCA-synthesis and therefore phenylalanine serves as an ideal and straightforward test bed. To the best of our knowledge, in situ formation and grafting of hydrophobic organo-gels within an inorganic porous environment has not been studied so far. As an inorganic substrate, sintered porous alumina membranes were prefunctionalized with (3-aminopropyl)trimethoxysilane (APTMS) to enable surface-initiated polymerization. The polymerization of PA-NCA was performed in solvent mixtures of THF and DCM, employing different volume fractions of these solvent mixtures. We mainly investigate the morphology of the grafted polypeptitde films and their structural stability after a treatment with a mixture of chloroform and dichloroacetic acid (CHCl_3_/DCA). The observed chemical and structural stability of the grafted polymer films is important for long-term applications. We believe that the increase of active surface of supported organo-gels of such composite membranes can be further improved with regard to adsorption materials in medical and analytical applications or analytical separation processes.

## Results and Discussion

**Figure 1:**
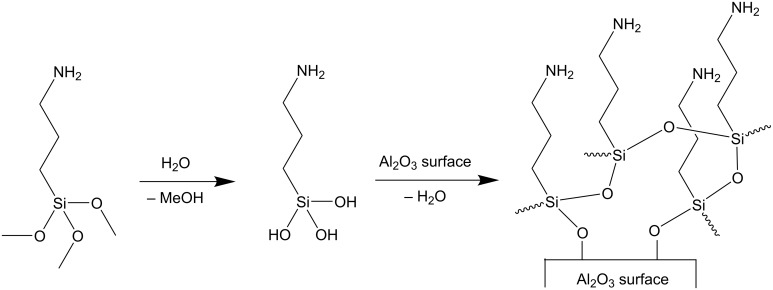
Hydrolysis of methoxy groups and functionalization of the surface of the ALOX-membrane due a condensation reaction between hydroxyl groups on the surface and hydroxylated silanes.

Inorganic porous membranes of sintered corundum particles (ALOX-membranes) were used as substrates for polymer functionalization [33–34]. The surface of ALOX-membranes contains hydroxyl groups, which can be used for chemical modification [35]. Their porosity predetermines different conditions in wet chemical surface functionalization due to a restricted and diffusion limited transport of reactants to the inner surface of the ALOX-membrane. For this reason, we distinguish between the inner and the outer surface of this porous substrate (see Supporting Information File 1, Figure S1). The average pore diameter of the ALOX-membranes was determined by Hg-porosimetry measurements to 1348 nm (see Supporting Information File 1, Figure S2). The surface of the ALOX-membranes was prefunctionalized by silanization to form an initial layer of initiating groups on the surface of the ALOX substrate (see Figure 1). As initiators, primary amino groups were used, which were immobilized by covalent bonding of amino groups containing silanes such as APTMS [36]. For an effective grafting process, first a hydroxylation of the methoxy groups of APTMS is beneficial which can be induced by adsorbed water on the ALOX surface [37]. Trifunctional organosilanes such as APTMS enable polycondensation and the formation of a vertically polymerized and dense layer of amino silanes (see Figure 1) [36].

**Figure 2:**
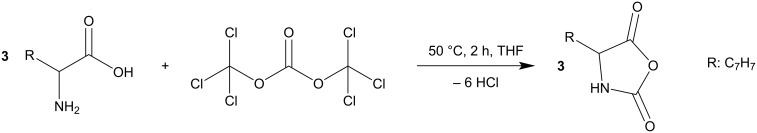
Synthesis of NCA-monomers from α-amino acids and triphosgene.

A "grafting-from" process of the hydrophobic polymers was then achieved by surface-initiated ring-opening polymerization (SI-ROP) of activated NCA-monomers. NCA-monomers were synthesized via a modified Fuchs–Farthing method, employing the reaction of DL-phenylalanine with triphosgene (see Figure 2) [38–39].

**Figure 3:**
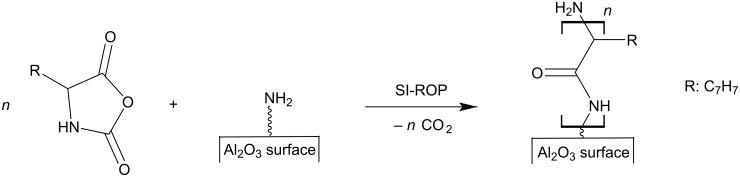
Surface-initiated ring-opening polymerization of NCA-monomers.

The following polymerization of the NCA-monomers is initiated by the primary amino groups of the immobilized silanes at the surface of the porous ALOX-membrane (see Figure 3) in solvent mixtures consisting of different volume fractions of THF and DCM.

The functionalized ALOX-membranes were subsequently washed with fresh solvent mixtures followed by a treatment with a mixture of chloroform (CHCl_3_) and dichloroacetic acid (DCA) (80/20 vol %) to remove weakly bonded polymers [13]. Noncovalently bonded polymer chains can result from hydrolyzed monomers or an alternative polymerization mechanism [40–41]. The characterization of these composite materials was performed by NIR-spectroscopy (NIR), water contact angle measurements (CA), scanning electron microscopy (SEM) and thermogravimetric (TG) measurements. Mid- and near-infrared spectroscopic analysis provides qualitative information of the secondary structure of the grafted poly(amino acids). The measurement of the water contact angle allows conclusions regarding the hydrophobic or hydrophilic properties of the functionalized surface as well as the microstructure of the grafted polymer films at the outer surface. Thermogravimetric measurements were used to determine the amount of strong and weakly bonded polymers inside the ALOX-membrane. Finally, SEM was used to investigate the microstructure and morphology of the grafted polymers at the outer surface as well the inner surface of the ALOX-membranes.

Previous experiments showed a significant influence of the used solvent on the microstructure of the grafted polyphenylalanine (pPA) within the pore volume of the macroporous ALOX-membranes. The polymerization of 100 mM pPA-NCA monomer solutions in pure THF results in a flat and smooth polymer film, whereas polymerization in pure DCM leads to the formation of a three-dimensional network of agglomerated fibrils that expand throughout the full pore volume of the porous ALOX-membrane (thus clogging its interior). In this study, we investigate the influence of different mixtures of THF and DCM on the microstructure and the amount of grafted pPA-agglomerates at the outer surface and the inner surface of the functionalized ALOX-membranes. SI-ROP was performed in solvent mixtures with fractions of 0, 50, 75, 90 and 100 vol % of dichloromethane in THF/DCM mixtures at room temperature for 24 hours.

### SEM characterization and contact angle measurements of pPA-functionalized ALOX-membranes

**Figure 4:**
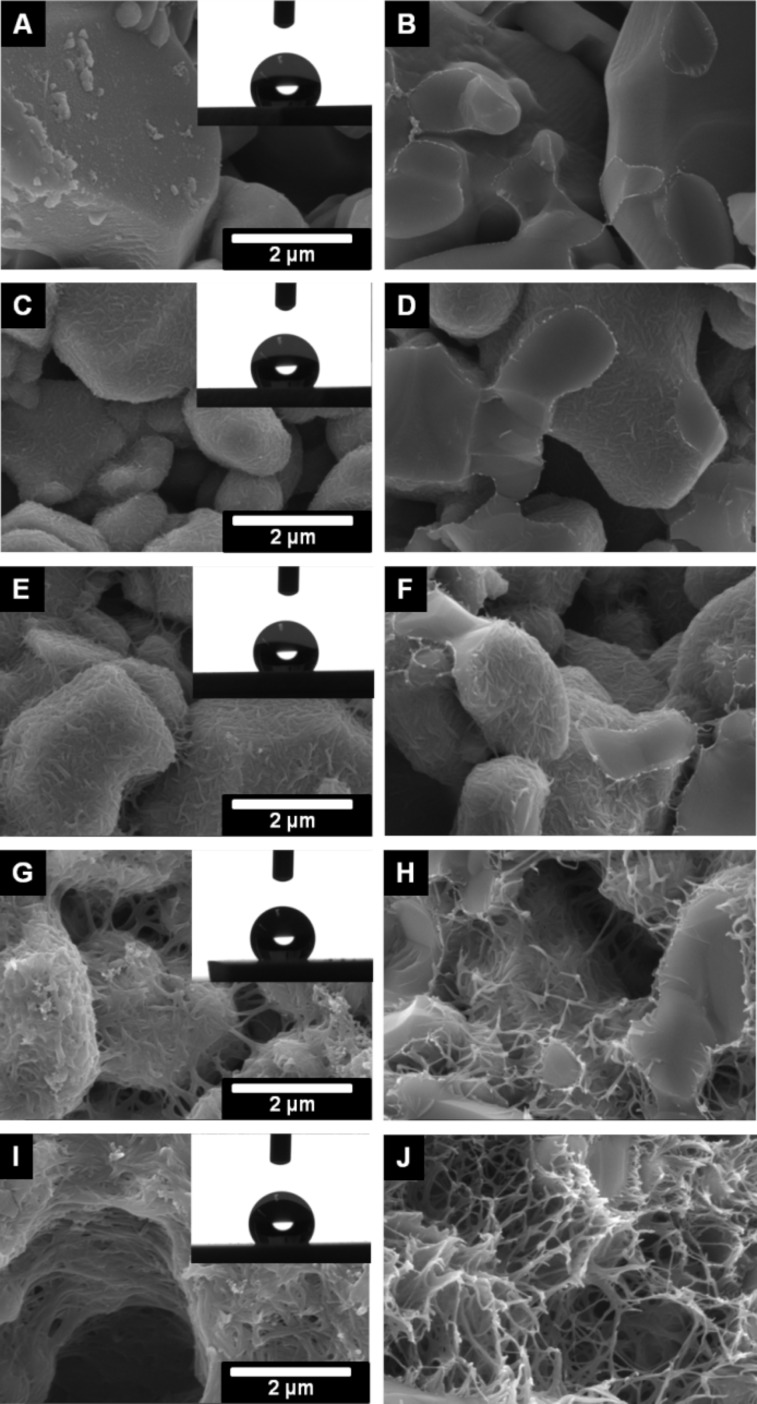
SEM images of the outer surface (A, C, E, G and I) and inner surface (B, D, F, H, J) of the polyphenylalanine functionalized ALOX-membranes from solvent mixture with 0 vol % (A and B), 50 vol % (C and D), 75 vol % (E and F), 90 vol % (G and H) and 100 vol % (I and J). Inserts represents the water contact angle (2 µL drop size).

**Figure 5:**
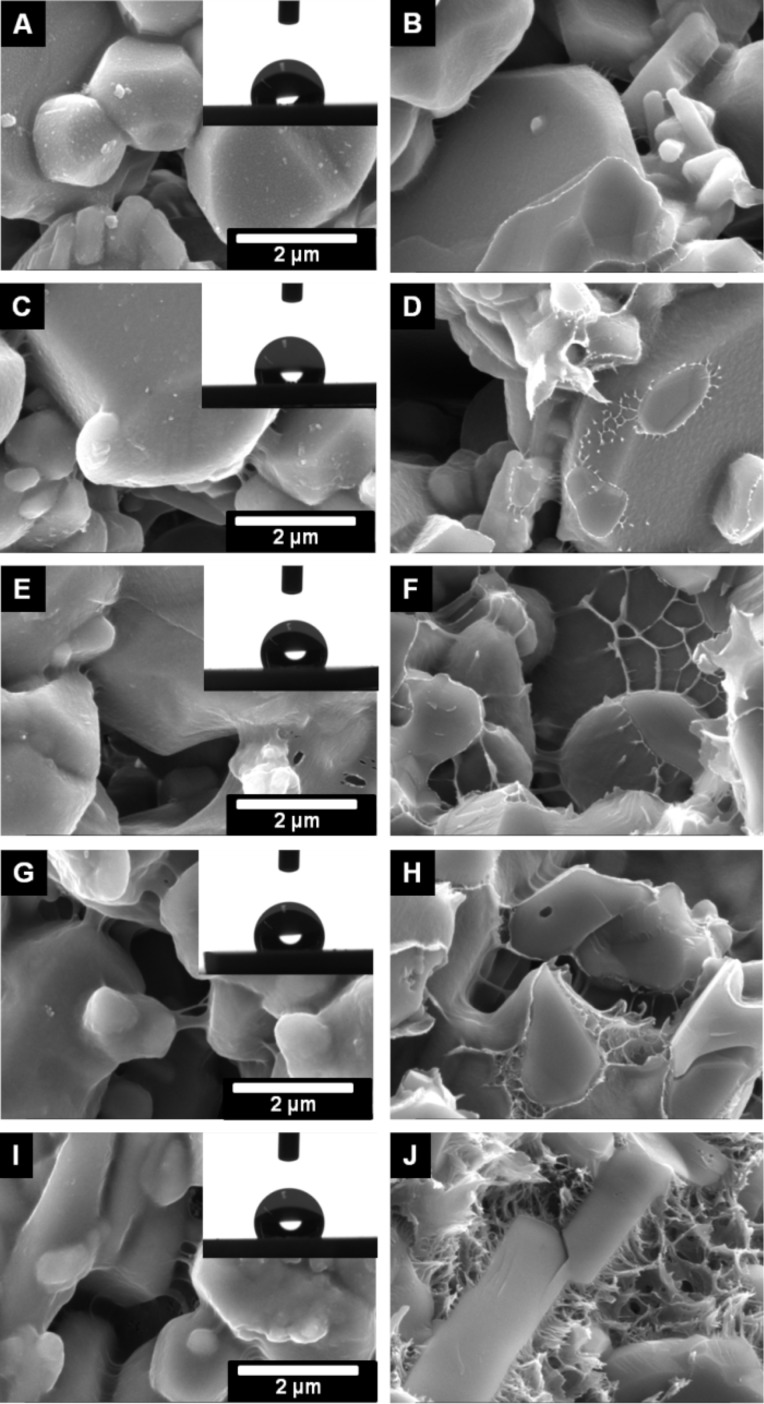
SEM images of the outer surface (A, C, E, G and I) and inner surface (B, D, F, H, J) of the polyphenylalanine functionalized ALOX-membranes from solvent mixture with 0 vol % (A and B), 50 vol % (C and D), 75 vol % (E and F), 90 vol % (G and H) and 100 vol % (I and J) after treatment with CHCl_3_/DCA. Inserts show the water contact angles with a volume of 2 µL.

The analysis of the SEM results shows a significant change of morphology of the grafted polymer films (see Figure 4) due to the use of different solvent mixtures. The observed surface roughness and the amount of formed fibrillar structure increases with increasing volume fraction of DCM. The polymerization of PA-NCA in pure THF typically results in a flat and smooth polymer film, which is barely observable even via SEM. Nevertheless, the water contact angle is significantly increased to 125.4 ± 2.8° compared to the neat ALOX-membrane before functionalization. Thus, the outer surface of the membrane shows a strong hydrophobic character due functionalization with polyphenylalanine. This observation can be related to the hydrophobic character of the side chain of the phenylalanine monomer units. Neat ALOX-membranes are highly hydrophilic and show no water contact angle (see Supporting Information File 1, Figure S3). The contact area of neighboring corundum particles at the inner surface of the porous ALOX-membrane is clearly visible and marks the functionalized and unfunctionalized alumina surface regions (see Figure 4 and Figure 5). A content of 50 vol % of DCM results in the formation of rod-shaped structures on the functionalized surface (see Figure 4C and 4D). The main axis of these rod-shaped structures is plane-parallel to the surface of the corundum particles. These structures can be associated with a bundle of agglomerated α-helices of polyphenylalanine, which were formed during polymerization and/or dewetting during the drying process after polymerization. Here no significant difference in morphology of the polymer film at the functionalized areas at the outer and the inner surface can be observed. Nevertheless, the contact angle is further increased to 130.9 ± 0.1°. With increasing volume fraction of DCM in the solvent mixture, the length and thickness of the rod-shaped structures increases. In addition, a difference in morphology of the polymer structures at the outer and inner surface become observable. At the outer surface, the rod-shaped structures show an increase of thickness and the orientation of their main axis remain plane-parallel to the surface of the corundum particles. This is in contrast to the morphology of the polymer films at the inner surface which expands increasingly into the pore volume of the ALOX-membrane. These observations can be explained by a stabilizing effect from nearby corundum particles, which enables a second contact site for the growing polymer chains. In addition, growing polymer chains can interact with polymer chains, which are growing from the opposing surface region, due to intermolecular interactions. We assume that by increasing the volume fraction of DCM an alternative polymerization mechanism (activated monomer mechanism or carbamat mechanism) takes over, which enables free growing polymer chains and/or polycondensation reactions [42–46]. In the activated monomer mechanism, the N–H proton is removed from the NCA-monomer by a base (e.g. tertiary amines), leading to the formation of a NCA-anion. We assume that the amino groups in the amino silane film may act as sterically hindered tertiary amines. The resulting NCA-anion reacts as a nucleophile and initiates an ROP, which leads to a growing polymer chain with two functional end groups in solution. The intermolecular and intramolecular coupling of the NCA-cyclic end with the corresponding terminal amine group results in polycondensation and cyclization, respectively. Furthermore, in the carbamate mechanism the intermediate carbamic acid is stabilized by forming the corresponding carbamic acid salt. Ballard et al. pointed out that the carbamic acid salt catalyzes the propagation of the ROP and results in higher rates of chain growth in contrast to the amine mechanism [45,47–48]. The degree of carbamic acid salt formation depends on factors like used solvents or CO_2_ removal during polymerization [49]. Concerning the role of the underlying alumina substrate, we speculate that the CO_2_ removal within the porous environment by diffusion is very limited and therefore the formation of carbamic acid salt is likely. During dewetting, the resulting polymer chains agglomerate and form the observed supramolecular structures, which are connected to multiple sites on the inner surface of the corundum particles (see Figure 4H and 4J). Therefore, polymer chains at the outer surface agglomerate, but collapse during the dewetting process because no mechanical stabilization from neighboring corundum particles is present. Therefore, the ability to form fibrillar structures can be associated with an increase of the amount of free polymer chains as well as an increase of solubility of the formed polyphenylalanine chains with an increase of the volume fraction of DCM during polymerization. Kricheldorf et al. have demonstrated that certain parameters such as solvent, the kind of initiator and monomer/initiator ratio directly affect the molecular weight and secondary structure of polyphenylalanine [50]. However, the polymerization of PA-NCA in pure DCM leads to a three-dimensional network of fibrillar bundles within the pore volume of the prefunctionalized ALOX-membrane. The stabilizing effect of the surrounding pore system is obvious and shown in Figure 4I and 4J. The participation of polyphenylalanine in solution does not show any fibrillar structures (see Supporting Information File 1, Figure S4).

**Figure 6:**
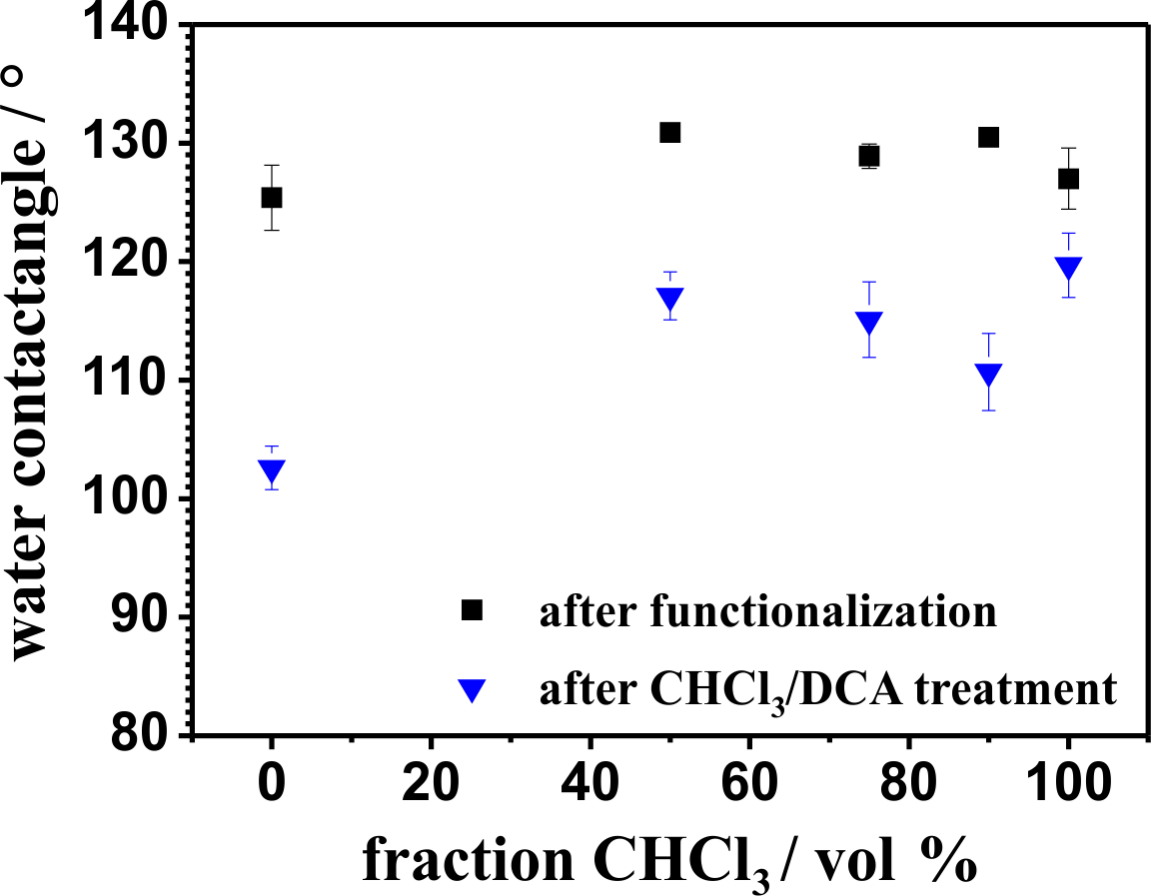
Water contact angles at the outer surface of the polyphenylalanine functionalized ALOX-membranes after the grafting process (black squares) from different solvent mixtures and after a CHCl_3_/DCA treatment (blue triangles).

We also studied the amount of weakly bonded polymers by post-treatment of the pPA functionalized ALOX-membranes in a mixture of chloroform and dichloroacetic acid (80/20 vol %). It has been shown that such mixtures can remove physically adsorbed polypetides on flat surfaces [51]. The treatment was performed by soaking the membranes in the mixture for 18 hours at room temperature. Diffusion limited dissolution of the weakly bonded polymers is the main process of removal of the adsorbed polymers at the inner surface although frequently shaking was performed to enhance the process. Therefore, a distinction between the outer and inner surface has to be considered. A clear deformation of the morphology of the polymer structures is observed after the treatment with the CHCl_3_/DCA mixture. Interestingly, at the outer surfaces, the former rough morphology turns into a smooth morphology, resulting in a decrease of the water contact angle (see Figure 5 and Figure 6).

This observation can be attributed to the removal of weakly bonded polymers from the outer surface or a rearrangement of the secondary, tertiary, or quaternary structure of the polypeptides. The reduction of the measured water contact angle can be explained by the established model of Cassie and Baxter, in which the wetting properties are affected by heterogeneous surfaces (inner surface is not wetted) and the roughness of the outer surface [52–54].

[1] $$\cos\theta_{\text{rough}}=r_{f}f\cos\theta_{\text{smooth}}+f-1$$

where θ is the Young contact angle on smooth surfaces, *r_f_* is the roughness ratio, which is defined by the ratio of the true surface area and the apparent surface area of the solid–liquid interface, and *f* is the fraction of the projected area of the solid that is wetted by the liquid. Equation 1 shows that the Young contact angle on smooth and hydrophobic surfaces (θ > 90°) is further increased by increasing the surface roughness ratio *r_f_*. If the fraction of the projected area of the outer surface (which is wetted by the water droplet) becomes about 100% (*f* = 1), the model of Wenzel can be applied [55–57]. Here we assume a relatively high fraction, *f*, because no roll off angles or high adhesion properties of the water droplets are observed (see Supporting Information File 1, Figure S5).

The morphology of the polymer structure within the pore volume is strongly affected by treatment with CHCl_3_/DCA. We assume that the polymeric structure within the pore volume swells due to interaction with the CHCl_3_/DCA mixture. In this swollen state, the agglomerated pPA-α-helices are able to change the secondary, tertiary and quaternary structure with subsequent rearrangement during the second dewetting process. At the outer surface the ability to rearrange is improved by convection initiated due to frequent mechanical agitation of the flask during the CHCl_3_/DCA treatment.

### NIR characterization of grafted polyphenylalanine

**Figure 7:**
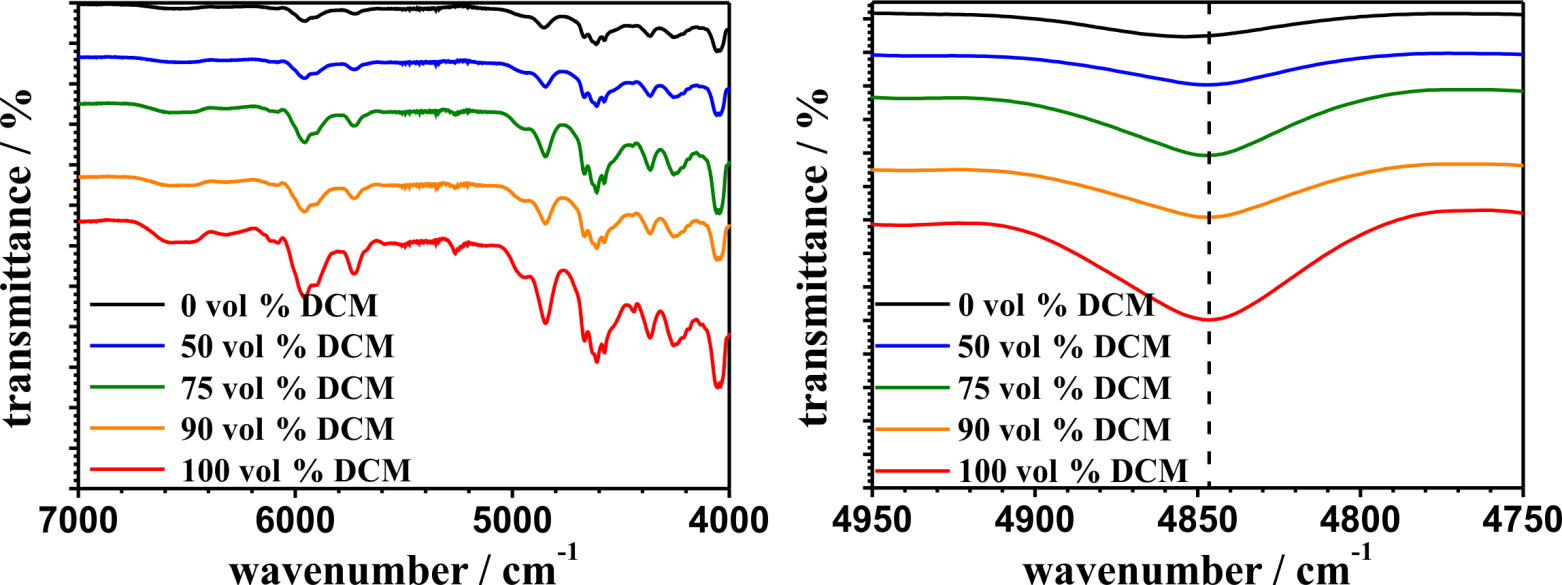
NIR spectra of the polyphenylalanine functionalized ALOX-membranes obtained from different solvent mixtures at different wavenumber regions after the grafting process. The dotted line indicates the maxima of the combination band at 4847 cm^−1^ of the polyphenylalanine functionalized membrane from pure DCM.

Near-infrared spectroscopic analysis can provide additional information about the secondary structure of the grafted polypeptides on the outer surface. Usually secondary structure of peptides can be easily detected by IR due the position of the amide I band (C=O) [50–51,58–63]. However, a proper contact of the functionalized ALOX surface and the crystal of the ATR module could not be achieved, therefore we employed diffuse reflectance infrared Fourier transform spectroscopy (DRIFT) to analyze surface-grafted polymers. Figure 7 shows the NIR spectra of the outer surface of the pPA-functionalized ALOX-membranes.

All observed NIR-bands can be assigned to the grafted polyphenylalanine chains [64] (Table 1).

**Table 1:** Assignment of NIR bands of polyphenylalanine grafted on ALOX [64].

NIR bands/cm^−1^	Assignment

6571	first overtone NH stretching mode
5955	first overtones CH (aromatic) stretching modes
5730	first overtones CH (aliphatic) stretching modes
4855–4847	combination amide A + amide II modes
4667	combination CH (aromatic) stretching + bending mode
4628	combination CH (aromatic) stretching + bending mode
4613–4610	combination amide B + amide II modes
4575	combination CH (aromatic) stretching + bending mode
4365	combination CH (aliphatic) stretching + bending modes
4256	
4056	combination CH_2_ stretching + CH_2_ rocking mode

The NIR band of ALOX assignable to modes from adsorbed water vanishes after pPA-functionalization (see Supporting Information File 1, Figure S6) [65–67]. The spectra of the different pPA-functionalized membranes are very similar except a minor red shift of the bands around 4855 cm^−1^ and 4613 cm^−1^ with increasing DCM volume fraction in the solvent mixture (see Figure 7 and Supporting Information File 1, Figure S7). These bands are assigned to combinations of amide A and amide II with amide B and amide II modes, respectively [64]. Whereas the amide A mode is assigned to an intermolecular hydrogen-bonded NH stretching vibration, the amide B band is due to the "free" NH stretching vibration and amide II to the NH bending motion of the peptide bond. Schultz et al. demonstrated a correlation between the amide I band (C=O stretching vibration) at 1641 cm^−1^ (indicating a β-sheet conformation) in the MIR region and the combination band of amide A and amide II in the NIR region at 4867 cm^−1^ [68]. The authors observed an increase of intensity as well a small red shift of the combination band between 4890 and 4860 cm^−1^ with increasing intensity of the amide I mode at 1641 cm^−1^. In accord with these observations we assign the observed red shift of the maximum of the combination band of amide A and amide II to an increasing amount of β-sheet conformations.

**Figure 8:**
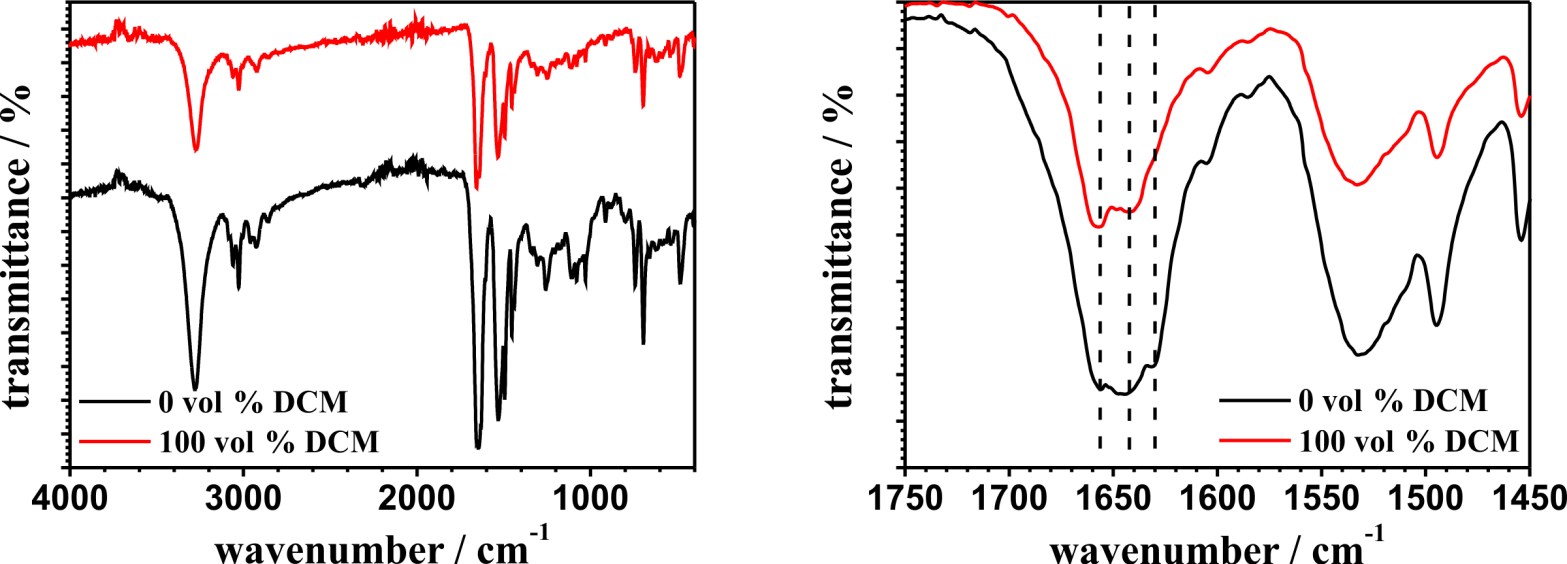
MIR spectra of polyphenylalanine synthesized from solution (pure THF black line and pure DCM red line) at different wavenumber regions. Dotted lines indicate typical values of the amide I vibration band for α-helix (around 1665 cm^−1^), random coil (between 1645–1630 cm^−1^) and β-sheet (around 1630 cm^−1^) conformations.

The maximum of the combination band ν (amide A + amide II) from the pPA-functionalized ALOX-membrane, which is functionalized in pure THF is located at 4855 cm^−1^ whereas the maxima of the ALOX-membranes, which were functionalized with DCM, are localized at 4847 cm^−1^ (see Table 1). This observation indicates a secondary structure of mostly β-sheet conformation within the polymer structure, formed in pure THF. The MIR spectra of in-solution-initiated free polymer chains of polyphenylalanine provide more detailed information about the secondary structure of the polymers, which were synthesized in different solvents. Figure 8 shows the MIR spectra of polyphenylalanine, which is synthesized in pure THF and pure DCM. The initiation in solution was performed using 1 mol % of *n*-butylamine with respect to pPA-NCA (100 mM).

**Figure 9:**
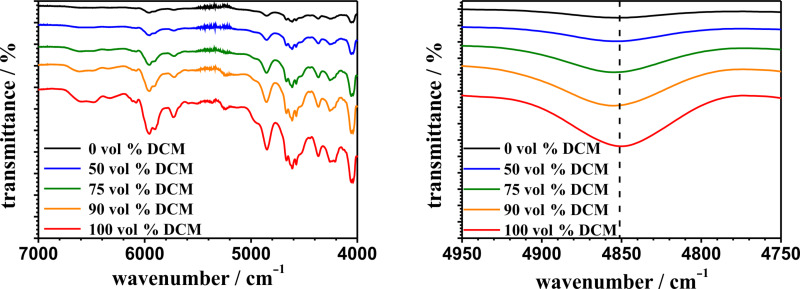
NIR spectra of the polyphenylalanine functionalized ALOX-membranes from different solvent mixtures at different wavenumber regions after treatment with CHCl_3_/DCA. Dotted lines indicate the maxima of the combination band at 4850 cm^−1^ of the polyphenylalanine functionalized ALOX-membrane from pure DCM.

The MIR spectra of polyphenylalanine synthesized in pure THF and pure DCM show a broad amide I band in the region between 1700 and 1600 cm^−1^. The absorption maxima at wavenumbers around 1656, 1645 and 1632 cm^−1^ are assigned to α-helix, random coil and β-sheet conformations, respectively. The MIR spectra confirm a dominant α-helix structure in pPA, which was synthesized in pure DCM, whereas the MIR spectrum of pPA, which was synthesized in pure THF, confirms an increasing content of β-sheet and random coil conformations. The combination band around 4613 cm^−1^ (amide B and amide II) in the NIR spectra (see Supporting Information File 1, Figure S7) also shows a small red shift with increasing volume fraction of DCM. We assume that these shifts are also correlated to the content of β-sheet conformations in the same way as the combination band around 4855 cm^−1^ is observed [68–69]. Therefore, NIR analysis provides information on the secondary structure of grafted polypeptides due to the shift of combination bands, in which NH stretching and NH bending modes are involved. Nevertheless, the information is still very limited in comparison to MIR spectroscopic analysis. Figure 9 shows the NIR spectra of the pPA-functionalized ALOX-membranes after treatment with the CHCl_3_/DCA mixture.

After treatment with CHCl_3_/DCA both combination bands (amide A + amide II and amide B + amide II) appear slightly broadened. In addition, the location of the maxima of these bands converge as shown in Table 2.

**Table 2:** Assignment of the combination bands with NH-stretching and NH-bending modes involved for different polyphenylalanine functionalized ALOX-membranes.

Fraction CHCl_3_ / vol %	Amide A + Amide II / cm^−1^	Amide B + Amide II / cm^−1^	Assigned to dominant secondary conformation

after functionalization			

0	4855	4613	α-helix/β-sheet/random coil
50	4847	4611	α-helix/random coil
75	4847	4611	α-helix/random coil
90	4847	4611	α-helix/random coil
100	4847	4610	α-helix/random coil

after CHCl_3_/DCA treatment			

0	4852	4613	random coil
50	4853	4613	random coil
75	4854	4613	random coil
90	4855	4613	random coil
100	4850	4611	random coil

This observation is a result of the removal of weakly bonded polymer chains as well as a rearrangement of the secondary, tertiary and quaternary structure of the polypeptides at the outer surface of the ALOX-membrane. We assume that the treatment with the CHCl_3_/DCA mixture and the subsequent second dewetting process results in a transition of secondary structures which were formed during the first dewetting process after polyphenylalanine functionalization. These results demonstrate that a former dominant secondary structure (β-sheets for 0 vol % DCM and α-helix for 50–100 vol % DCM) transforms to a more random coil-like conformation after treatment with CHCl_3_/DCA.

### Thermogravimetric measurements of the inorganic–organic composite membranes

**Figure 10:**
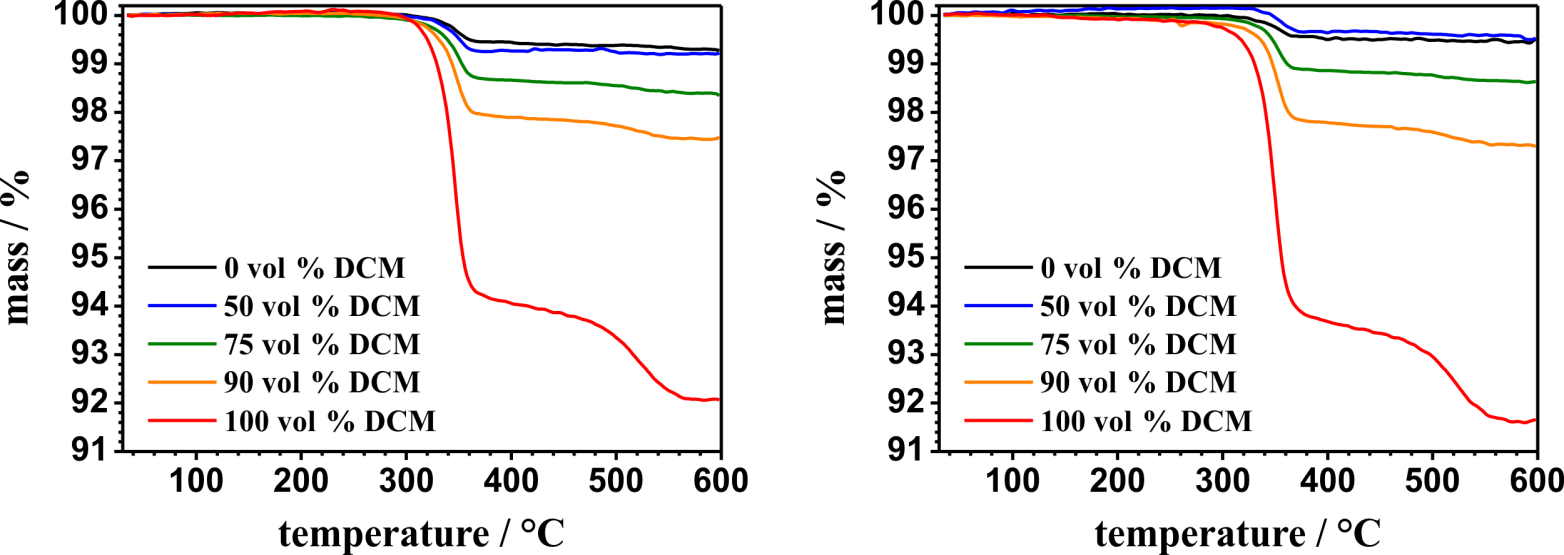
Thermogravimetric measurements from differently functionalized polyphenylalanine ALOX-membranes after the grafting process (left diagram) and after CHCl_3_/DCA treatment (right diagram).

We also investigate the amount of weakly bonded polymer chains using thermogravimetric analysis in order to see whether the change of morphology as well as the change of dominant secondary structure is a result of the removal of weakly bonded polymer chains or simply affected by the rearrangement of grafted polymer chains. Figure 10 shows the thermogravimetric measurements of different polyphenylalanine functionalized ALOX-membranes.

**Figure 11:**
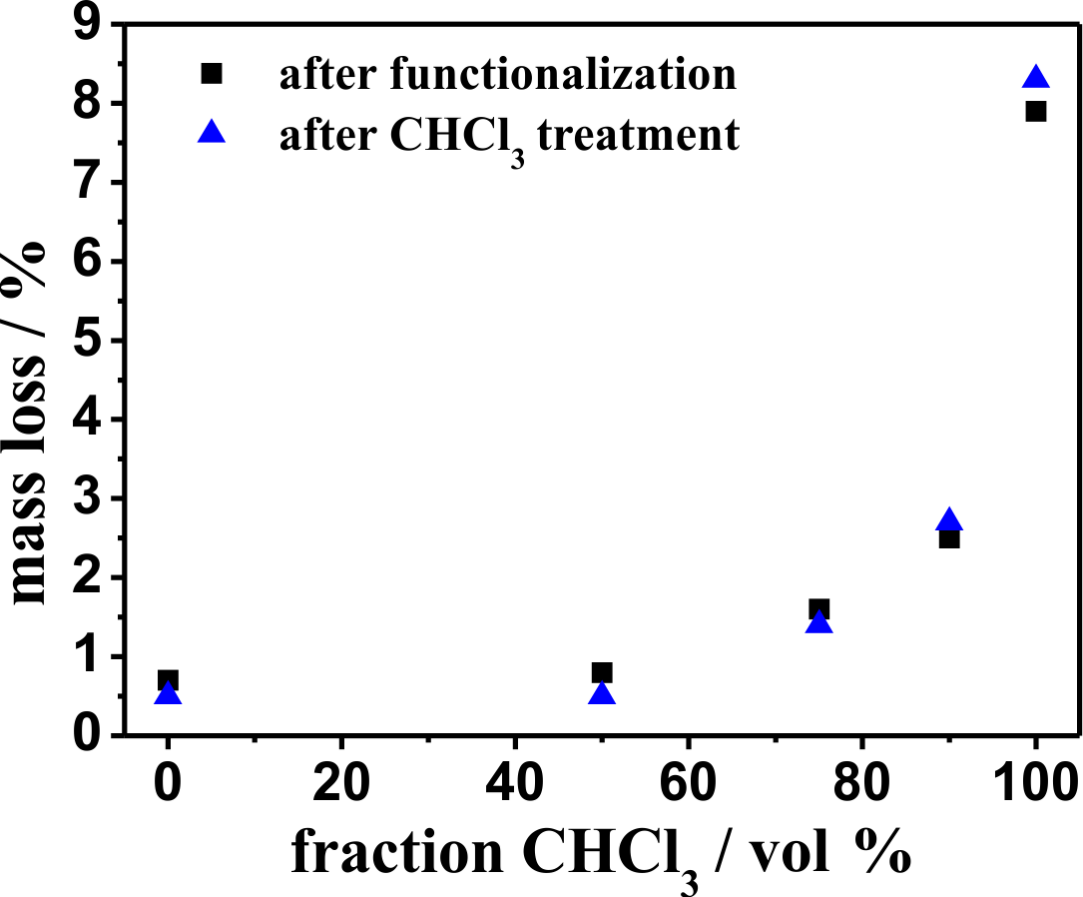
Relative mass loss after heating to 600 °C under air atmosphere for different polyphenylalanine functionalized ALOX-membranes.

The thermal analysis in oxygen atmosphere showed a first decay step after 310 °C and a second decay step after 490 °C. The total value of the relative mass loss was determined by simply subtracting 100% minus the mass (in %) at 600 °C. To calculate the amount of the removed polymer chains, the value of the relative mass loss is plotted against the volume fraction of DCM before and after the treatment with CHCl_3_/DCA (see Figure 11).

These results show that the amount of polymer does not change significantly after treatment with CHCl_3_/DCA which points to the fact that the amount of weakly bonded polymer chains is very low. As a result, the change in morphology of the observed polymer structures at the outer and inner surface of the porous ALOX-membranes as well as the change in secondary structure is assigned to a rearrangement process during the treatment with the CHCl_3_/DCA mixture and the subsequent second dewetting process exclusively.

### Adsorption experiments

**Figure 12:**
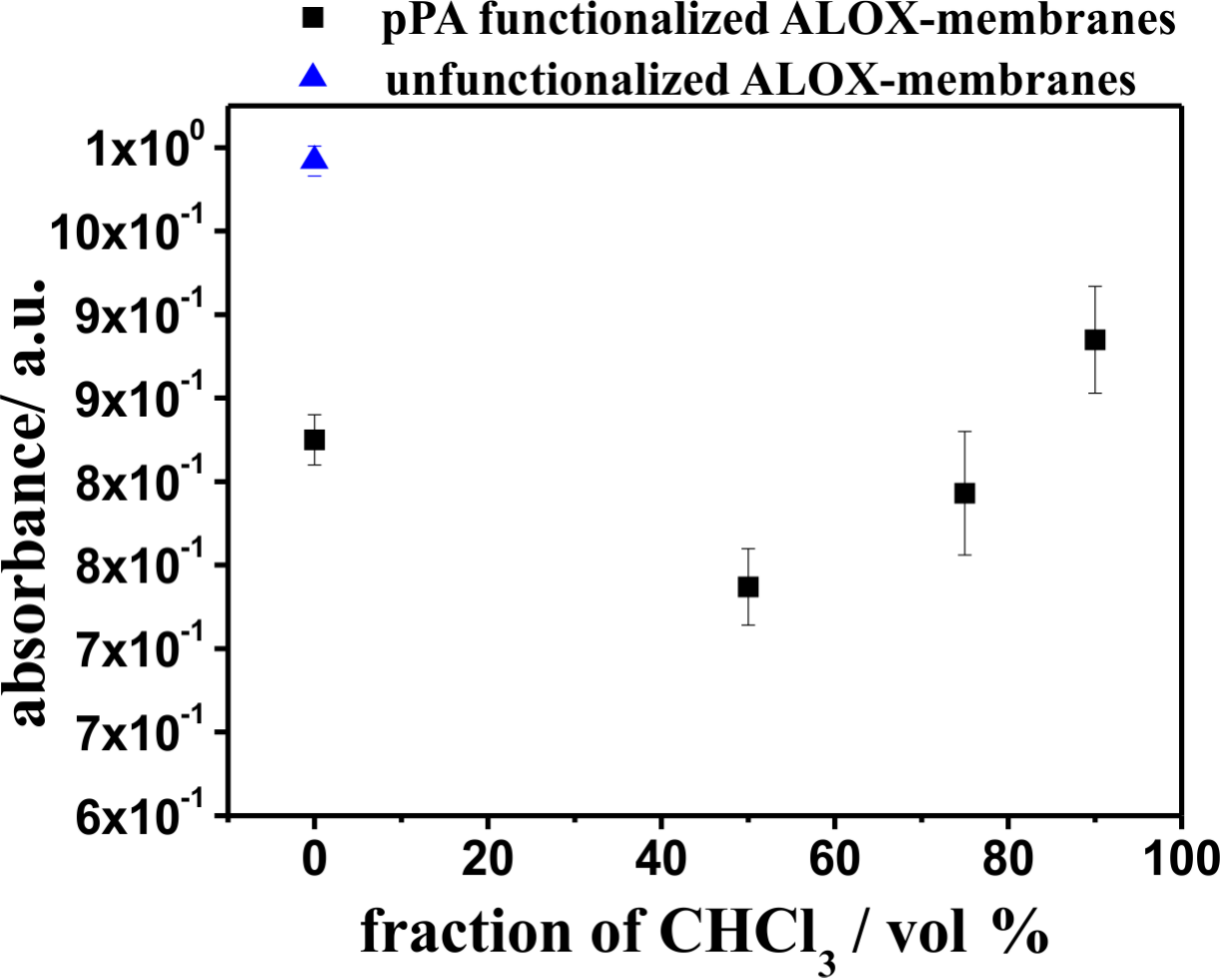
Absorbance values of the water fractions with chloroanilic acid after the loading step.

Further experiments were performed to investigate the adsorption capability of the organic–inorganic composite membranes with regard to possible later use in reversed-phase solid phase extraction applications [70–72]. As test the analyte we used the slightly water-soluble and UV–vis-detectable chloroanilic acid [73]. Chloroanilic acid was dissolved in deionized water and the solution was pumped through the porous composite membranes using a syringe pump. Subsequently, the concentration of chloroanilic acid in the collected solution was determined with UV–vis-spectroscopy. The amount of adsorbed chloroanilic acid was calculated from the absorbance of the analyte stock solution (see Figure 12).

Unfunctionalized ALOX-membranes adsorb 16.7% of chloroanilic acid molecules, whereas pPA-ALOX-membranes, which were functionalized in THF (see Figure 4A and 4B), adsorb 30.8% of the analyte molecules. It is observed that the hydrophobic pPA film enhances the adsorption of chloroanilic acid. The adsorption capability of pPA-ALOX-membranes, which were functionalized in a solvent mixture of 50% dichloromethane (see Figure 4C and 4D), further increases to 38.1%. This observation can be assigned to a higher surface area due to the increased roughness of the grafted pPA polymer film. Nevertheless, the amount of adsorbed analyte molecules decreases when pPA ALOX-membranes are used, which were functionalized with a higher volume fraction of dichloromethane in the solvent mixture (see Figure 4E–H). Investigations of pPA-ALOX-membranes, which were functionalized in dichloromethane (see Figure 4I and 4J), show no effective flux of the analyte solution through the membranes. These observations indicate clogged pores and the absence of available flow channels through the functionalized ALOX-membranes. The decreasing capability of the pPA-ALOX-membranes, which were functionalized with 75 and 90% volume fraction of dichloromethane, can be explained due to the decreasing number of flow channels through the ALOX-membranes. Obviously, a further increase of the amount of grafted pPA polymers leads to a decreasing number of flow channels within the ALOX-membrane. Although we assume an increasing surface area with increasing amount of grafted polyphenylalanine (see Figure 4), the effective surface area, which is wetted during the adsorption experiments, is decreased by the decreasing amount of available flow channels. In fact, the increase of the surface area leads to a higher adsorption capability of the composite membranes. But at some point (between 0 and 50% volume fraction of DCM in the solvent mixture), the adsorption capability is decreased by a decreasing number of available flow channels. Owing to the remaining water in the elution fraction, the calculation of the amount of desorbed chloroanilic acid after the elution step with methanol is challenging. In this regard, the optimal conditions for effective use in reversed-phase solid phase extraction have to be further adjusted in future investigations.

## Conclusion

This study demonstrates the synthesis of inorganic–organic composite membranes, which may be useful as adsorption materials in medical and analytical applications. The influence of the volume fraction of DCM in the used solvent on the in situ formation of polyphenylalanine three-dimensional fibrillar networks within a macroporous environment could be demonstrated. The morphology of the observed polymer structures is strongly affected by the fraction of DCM in the solvent mixture. An increasing volume fraction of DCM in the reaction mixture results in an increase in the roughness of the grafted polymer film on the outer surface of the ALOX-membranes as well as in the inner three-dimensional network-like morphology of the polymer structures within the ALOX-membranes. Surface-initiated ring-opening polymerization of polyphenylalanine-NCA in pure DCM results in the formation of a three-dimensional network of agglomerated fibrils which fills the inner pores of the ALOX-membrane. It consists of predominantly α-helix conformations of the polymer chains whereas a grafting process in pure THF results in a smooth and flat polymer film with a mostly random coiled polymer conformation. Post-treatment of the grafted polymers with a solvent mixture of CHCl_3_/DCA results in the deformation of the morphology of the polymer structures. Thermogravimetric measurements confirm that the amount of weakly bonded polymer chains is negligible compared to the amount of grafted polymer chains and that the change in morphology is a result of an overall rearrangement of the polymers. NIR spectroscopic analysis reveals an increase of random coil conformations due the rearrangement of the polymer chains after the treatment with the CHCl_3_/DCA solvent mixture. Adsorption experiments suggest a potential use of such composite materials in reverse solid phase extraction applications. In summary, we have introduced a new approach to synthesize and stabilize hydrophobic xerogels within a macroporous inorganic substrate. We believe that this approach can help to achieve a deeper understanding of the formation of polypeptide gels, which can be used for adsorption materials or in analytical separation processes.

## Experimental

**General.** DL-Phenylalanine with purity of 99% was purchased from Sigma-Aldrich and used as received. Porous alumina ceramic membranes (ALOX-substrates) were purchased from Kerafol GmbH (Eschenbach, Germany) and laser cut to small discs with a diameter of 13 mm and a thickness of 0.5 mm. For the synthesis, dichloromethane and THF were dried by distillation over calcium hydride and sodium/benzophenone, respectively. For recrystallization *n*-pentane was dried by distillation over sodium/potassium eutectic alloy. SEM measurements were performed with an XL30 FEG (Philips) system. Contact angles and NIR-spectroscopic analysis were performed with DSA30 (Krüss GmbH) and Nicolet 6700 (Thermo Fisher Scientific) equipment, respectively, and thermogravimeric measurements were done with a TG209 F1 (Netzsch) system.

**Pretreatment and silanization of ALOX-membranes.** The ALOX-substrates were subsequently cleaned in boiling 35% H_2_O_2_/water solution and deionized water followed by drying at 100 °C in air over night. The ALOX-substrates were cooled to room temperature in air and stored under inert gas atmosphere until use. For silanization five ALOX-membranes were poured in 5 mL of a 1.5 vol % APTMS solution in dry toluene. The solvent was refluxed for 16 h under inert gas atmosphere followed by three washing steps with fresh toluene followed by drying under argon and under vacuum. Crosslinking of the grafted silanes was performed at 120 °C under vacuum for two hours. Amino-prefunctionalized ALOX-membranes were stored under inert gas atmosphere until use.

NH_2_-ALOX-membrane: IR (DRIFT), ν: 6477 bw, 5809 w, 5641 w, 4933 w, 4356 w, 4301 w cm**^−^**^1^.

**Synthesis of polyphenylalanine-NCA-monomer** [39]. 5 g (30.2 mmol) of DL-phenylalanine was carefully degassed in a dried 250 mL three-necked flask. The flask was connected to a gas bubbler and the outlet of the gas bubbler was connected with a washing bottle filled with 1 M NaOH solution of water. 50 mL of anhydrous THF and the suspension was warmed up to 50 °C. Under a stream of argon, 3 g (10.1 mmol) of triphosgene was added and the suspension was stirred for 20 min. The suspension starts to clear up and portions of 100 mg of triphosgene were added every 10 min until a clear solution was formed. After 1 h the solution was poured into anhydrous *n*-pentane under vigorous stirring. The resulting suspension was stored at −28 °C overnight to complete precipitation followed by filtration and repeated recrystallization from anhydrous THF and *n*-pentane. The colorless needles were filtered, washed with anhydrous *n*-pentane, dried under vacuum and stored under inert gas atmosphere at −28 °C until early use.

Phenylalanine-NCA: ^1^H NMR (500 MHz, THF-*d*_8_), δ: 2.86 (dd, 1H), 3.06 (dd, 1H), 4.48 (m, 1H), 7.17 (m, 5H), 7.80 (s, 1H) ppm. ^13^C NMR (125 MHz, THF-*d*_8_), δ: 37.63, 58.58, 126.97, 127.14, 128.45, 129.23, 135.87, 151.55, 170.07 ppm. IR (ATR), ν̃: 3281 s, 3064 w, 3028 w, 1833 m, 1797 w, 1770 s, 1497 w, 1366 m, 1303 m, 1295 m, 1116 m, 1096 w, 946 m, 916 s cm**^−^**^1^. MS (EI), *m*/*z*: 191 ([M]^+^). Melting point: 93–94 °C.

**Surface-initiated ring-opening polymerization of PA-NCA (grafting to process).** 96 mg (0.5 mmol) of PA-NCA was dissolved in 5 mL (total volume) of the corresponding solvent mixture composed of THF or DCM in a homemade flask, which allows orientation of the ALOX-membranes in an upright position during polymerization. Under inert gas atmosphere, three NH_2_-functionalized ALOX-Membranes were immersed in the PA-NCA solution for 24 h at room temperature. The functionalized ALOX-membranes were washed three times with 5 mL of fresh corresponding solvent mixture. The cleaning process was assisted by a 5 s treatment in a supersonic bath at each wash step. The functionalized ALOX-membranes were dried under argon atmosphere and vacuum and stored under argon atmosphere until use.

Polyphenylalanine-ALOX-membrane (100 vol % DCM): NIR (DRIFT), ν: 8757 w, 6580 bm, 5956 s, 5730 m, 5241 w, 4942 w, 4847 m, 4667 m, 4610 m, 4575 m, 4364 m, 4256 m, 4056 s, 4040 s cm**^−^**^1^.

**Synthesis of polyphenylalanine.** 96 mg (0.5 mmol) of PA-NCA was dissolved in 5 mL of anhydrous THF or anhydrous DCM in a homemade flask, which allows orientation of the ALOX-membranes in an upright position during polymerization. 50 µL of a 100 mM solution of *n*-butylamine in THF or DCM was added with subsequent mixing of the clear solution. The polymerization was performed for 72 h at room temperature without stirring. The formed CO_2_ could escape from a connected gas bubbler. After polymerization, the flask was opened and the solvent was removed by simple evaporation for 72 h. A white solid was obtained.

Polyphenylalanine (from DCM): NIR (ATR), ν: 3273 s, 3061 w, 3028 m, 2924 w, 1657 s, 1643 m, 1533 s, 1495 m, 1454 m, 1440 w, 743 m, 697 m, 486 m cm**^−^**^1^.

Polyphenylalanine (from THF): NIR (ATR), ν: 3280 s, 3061 w, 3028 m, 2961 w, 2928 w, 1656 s, 1645 s, 1533 s, 1495 m, 1454 m, 1441 w, 1259 m, 743 m, 695 m, 483 m cm^−1^.

**Adsorption analysis.** 2 mL of an aqueous solution of chloroanilic acid (48 µM) was pumped through the porous ALOX-membranes with a syringe pump at a flow rate of 1 mL/min. Prior conditioning of the ALOX-membrane was done by activation with 2 mL of methanol followed by equilibration with 2 mL of deionized water with the same flow rate. The amount of adsorbed chloroanilic acid (analyte) was determined from the collected solution of the loading step by UV–vis spectroscopy at a wavelength of 331 nm [73]. The membranes were placed in a stainless-steel filter holder from Millipore and sealed with silicone rings.

## Supporting Information

File 1 Additional experimental data.
