# Supplementary material for: A 3D-polyphenylalanine network inside porous alumina: Synthesis and characterization of an inorganic–organic composite membrane
Source: Beilstein J Nanotechnol. 2020 Jun 17;11:938–51. doi: 10.3762/bjnano.11.78 (PMC7308615; doi:10.3762/bjnano.11.78)
Supplement: File 1 — Additional experimental data. [file Beilstein_J_Nanotechnol-11-938-s001.pdf]

BEILSTEIN JOURNAL OF NANOTECHNOLOGY

Supporting Information

for

A 3D-polyphenylalanine network inside porous alumina: Synthesis and characterization of an inorganic–organic composite membrane

Jonathan Stott and Jörg J. Schneider

Beilstein J. Nanotechnol. **2020,** *11,* 938–951. doi:10.3762/bjnano.11.78

Additional experimental data

License and Terms: This is a supporting information file under the terms of the Creative Commons Attribution License (http://creativecommons.org/licenses/by/4.0). Please note that the reuse, redistribution and reproduction in particular requires that the authors and source are credited.
The license is subject to the *Beilstein Journal of Nanotechnology* terms and conditions: (https://www.beilstein-journals.org/bjnano)

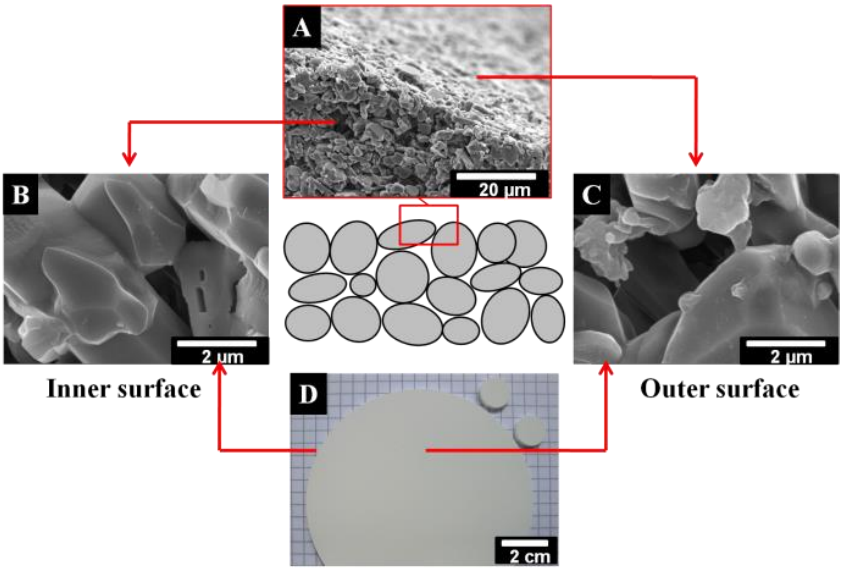

Figure S1:(A) SEM image of the edge of used ALOX-membranes. (B) SEM image of the inner surface, (C) SEM image of the outer surface and (D) photographimage of the ALOX-membranes. (Center) schematic of the ALOX-membrane morphology with their pore geometry.

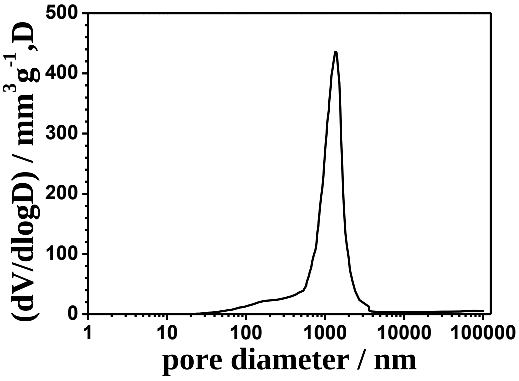

Figure S2: Hg-porosimetric measurement of a neat ALOX-membrane.

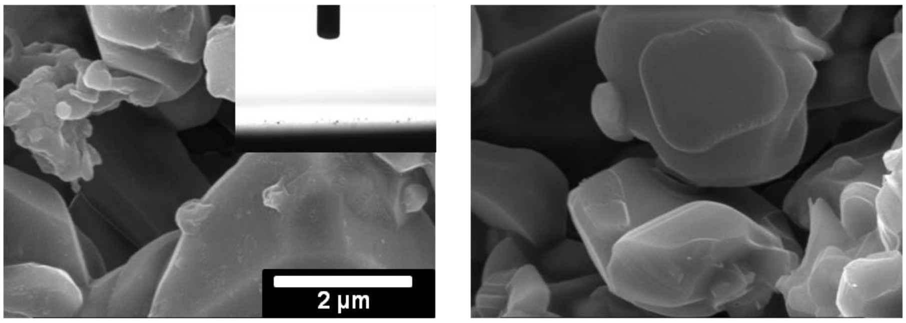

Figure S3: SEM image and water contact angle 2 µL drop size) of the outer surface of neat ALOX-membrane (left). SEM image of the inner surface of the ALOX-membrane.

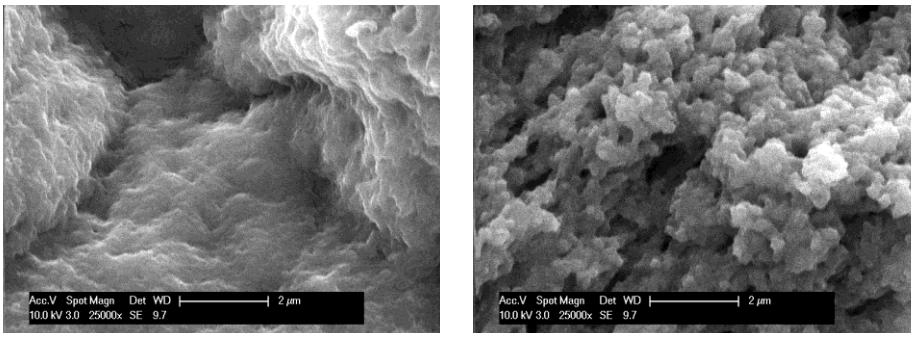

Figure S4:SEM images of polyphenylalanine synthesized in solution of pure DCM (left) and pure THF (right).

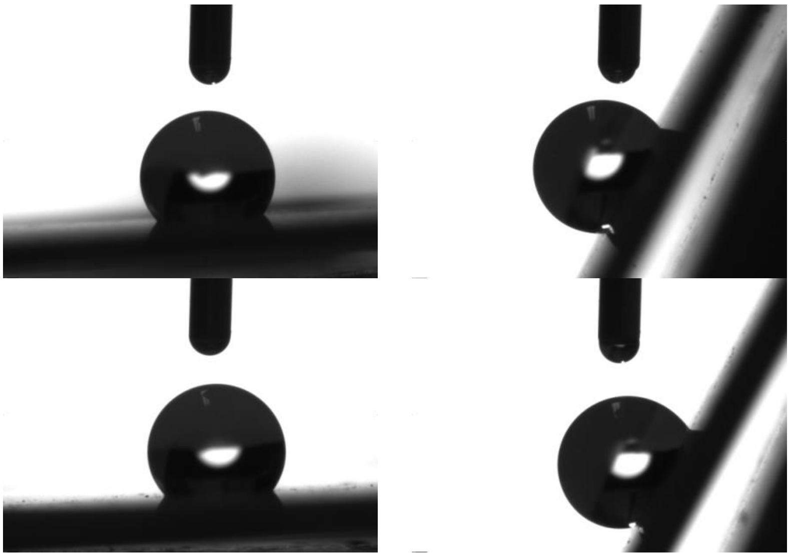

Figure S5: Water contact angles at flat orientation and tilted orientation of the pPA-functionalized ALOX-membrane.

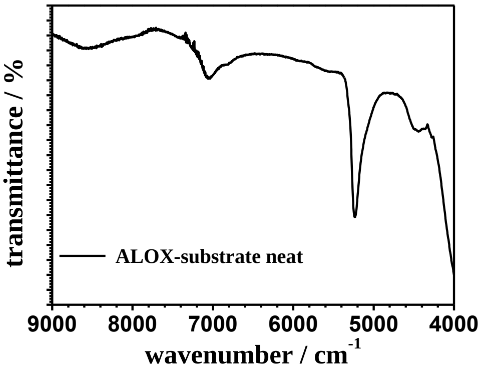

Figure S6: NIR-spectrum of neat ALOX-membrane (as achieved).

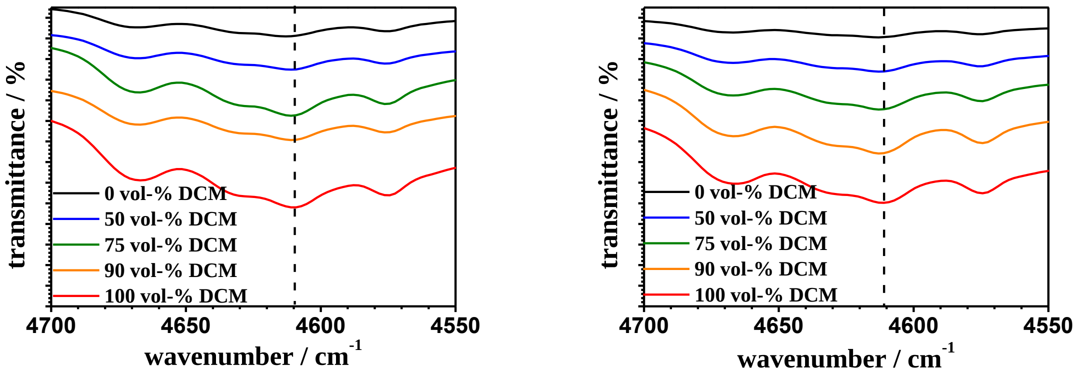

Figure S7: NIR spectra of the polyphenylalanine functionalized ALOX-membranes from different solvent mixtures before (left) and after (right) treatment with $CHCl_3$/DCA solvent mixture at different wavenumber regions after the grafting process. Dotted line indicates the maxima of the combination band at 4611 respectively 4610 cm^{-1} of the polyphenylalanine functionalized membrane from pure DCM.
